# Comparison of ARIMA and LSTM in Forecasting the Incidence of HFMD Combined and Uncombined with Exogenous Meteorological Variables in Ningbo, China

**DOI:** 10.3390/ijerph18116174

**Published:** 2021-06-07

**Authors:** Rui Zhang, Zhen Guo, Yujie Meng, Songwang Wang, Shaoqiong Li, Ran Niu, Yu Wang, Qing Guo, Yonghong Li

**Affiliations:** 1 Chinese Center for Disease Control and Prevention, Beijing 102206, China; zhangrui@chinacdc.cn (R.Z.); mengyj@chinacdc.cn (Y.M.); wangsw@chinacdc.cn (S.W.); lisq@chinacdc.cn (S.L.); 2 Institute of Medical Information and Library, Chinese Academy of Medical Sciences/Peking Union Medical College, Beijing 100020, China; guo.zhen@imicams.ac.cn; 3 National Institute for Nutrition and Health, Chinese Center for Disease Control and Prevention, Beijing 100050, China; niuran@ninh.chinacdc.cn; 4 National Institute of Environmental Health, Chinese Center for Disease Control and Prevention, Beijing 100021, China; wangyu@nieh.chinacdc.cn

**Keywords:** HFMD, ARIMA, ARIMAX, univariate LSTM, multivariate LSTM

## Abstract

Background: This study intends to identify the best model for predicting the incidence of hand, foot and mouth disease (HFMD) in Ningbo by comparing Autoregressive Integrated Moving Average (ARIMA) and Long Short-Term Memory Neural Network (LSTM) models combined and uncombined with exogenous meteorological variables. Methods: The data of daily HFMD incidence in Ningbo from January 2014 to November 2017 were set as the training set, and the data of December 2017 were set as the test set. ARIMA and LSTM models combined and uncombined with exogenous meteorological variables were adopted to fit the daily incidence of HFMD by using the data of the training set. The forecasting performances of the four fitted models were verified by using the data of the test set. Root mean square error (RMSE) was selected as the main measure to evaluate the performance of the models. Results: The RMSE for multivariate LSTM, univariate LSTM, ARIMA and ARIMAX (Autoregressive Integrated Moving Average Model with Exogenous Input Variables) was 10.78, 11.20, 12.43 and 14.73, respectively. The LSTM model with exogenous meteorological variables has the best performance among the four models and meteorological variables can increase the prediction accuracy of LSTM model. For the ARIMA model, exogenous meteorological variables did not increase the prediction accuracy but became the interference factor of the model. Conclusions: Multivariate LSTM is the best among the four models to fit the daily incidence of HFMD in Ningbo. It can provide a scientific method to build the HFMD early warning system and the methodology can also be applied to other communicable diseases.

## 1. Introduction

Hand, foot and mouth disease (HFMD) is common in children under five years old, but anyone can get it. The illness is usually not serious, but it is very contagious. It spreads quickly at schools and day care centers. HFMD has caused widespread social concern in countries such as China [1,2,3], Japan [4] and the United Kingdom [5]. Hence, exploring accurate prediction methods has great practical significance for the prevention and control of HFMD. Some studies have put forward different prediction methods for HFMD [6,7,8]. Time series analysis is a very powerful tool to detect disease status and predict future development, because it is based on the changes in historical datasets over time and produces mathematical models that can be extrapolated. Time series forecasting can be challenging as there are many different methods you could use and many different hyper parameters for each method [9]. Within the field of time series analysis, two models are particularly popular: the Autoregressive Integrated Moving-Average (ARIMA) model and the Long Short-Term Memory Neural Network (LSTM) model. The ARIMA model is one of the most popular methods used in infectious disease prediction, such as HFMD [2,8], COVID-19 [10], hepatitis [11], influenza [12], tuberculosis [13], as well as blood glucose concentrations and hypoglycemia [14], hospital daily outpatient visits [15], etc. LSTM is a special case of Recurrent Neural Networks (RNN) and is increasing in use in recent years in domains such as stocks [16], speech recognition [17], and disease prediction, such as HFMD [18], COVID-19 [19] and HIV [20].

Both ARIMA and LSTM are suitable for analyzing time series data and making predictions. For the prediction of the incidence of HFMD by the ARIMA model, most of the studies involve direct prediction [2,8], and some involve meteorological factors [21]. Simultaneously, most of the previously reported studies on HFMD prediction using the LSTM model were univariate, and were not combined with exogenous variables [18]. As far as we know, no studies have compared the accuracy of these two prediction models combined and uncombined with exogenous meteorological variables.

This study intends to identify the best model for predicting HFMD incidence in Ningbo by comparing ARIMA and LSTM models combined and uncombined with exogenous meteorological variables, based on the daily incidence of HFMD and daily meteorological data from 2014 to 2017 in Ningbo.

## 2. Materials and Methods

### 2.1. Study Area

Ningbo is located on the coast of the East China Sea and the southeast corner of the Yangtze River Delta. It is located in the Ningshao plain with moderate latitude (latitude from 28°51′ to 30°33′ N and longitude from 120°55′ to 122°16′ E). Its land area is 9714 km^2^, while its oceanic territory covers 9758 km^2^. At the end of 2017, the permanent resident population of Ningbo was 8.05 million. Ningbo belongs to the north subtropical monsoon climate zone, which is mild and humid, with obvious alternations of winter and summer monsoon. It has four distinct seasons, with four months in winter and summer and two months in spring and autumn. The annual average temperature of Ningbo is 16.4 °C, while the hottest (in July) is 28.0 °C, and the coldest (in January) is 4.7 °C. The annual average precipitation is 1480 mm. The annual average sunshine duration is 1850 h.

### 2.2. HFMD Incidence and Meteorological Data

The daily incidence data of HFMD from 1 January 2014 to 31 December 2017 in Ningbo were collected from the official website of The Data Center of China Public Health Science [22]. 

**Figure 1:**
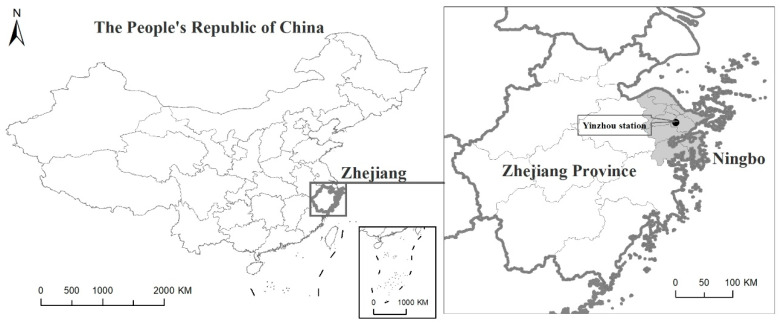
The geographical location of Ningbo and Yinzhou station.

The meteorological data during the study period were taken at Yinzhou station ($29^{{}^{\circ}}47^{’}\;N$, $121^{{}^{\circ}}33^{’}\;E$), the national meteorological monitoring station in the central city of Ningbo (See Figure 1). The meteorological data included daily mean temperature (Tmean, ℃), daily mean pressure (Pmean, hPa), daily mean relative humidity (RHmean, %), daily mean wind speed (WSmean,m/s), daily precipitation (PPTN, mm) and daily sunshine duration (Sunshine, h). These data were provided by the China Meteorological Administration.

### 2.3. Data Analysis

The data from January 2014 to November 2017 were set as the training set and the data of December 2017 were set as the test set.

The ARIMA and LSTM models with and without exogenous meteorological variables were adopted to fit the training set of the data for predicting the testing set of the daily incidence of HFMD.

All the four models were combined with “Rolling Forecast”. We predicted just one day; we took into account the real value on that day, and then we predicted the next day, etc.

Three indexes were selected as the measures to evaluate the performance of the models. 

The first performance measure is root mean square error (RMSE), which is used to compare the predicted value with the actual value. The RMSE is computed as:

$$\mathrm{RMSE}=\sqrt{\frac{\sum_{i=1}^{n}{\left(X_{i}-{\hat{X}}_{i}\right)}^{2}}{n}}$$

The second performance measure is mean absolute error (MAE). The MAE is defined as:

$$\mathrm{MAE}=\frac{\sum_{i=1}^{n}\left|X_{i}-{\hat{X}}_{i}\right|}{n}$$

The third performance measure is mean absolute percentage error (MAPE), a measure of relative overall fitness. This performance measure is defined as:

$$\mathrm{MAPE}=\frac{\sum_{i=1}^{n}\frac{\left|X_{i}-{\hat{X}}_{i}\right|}{X_{i}}\times 100}{n},$$

$X_{i}$ is the observed daily incidence of HFMD on the *i* day, and ${\hat{X}}_{i}$ is the predicted daily incidence of HFMD on the *i* day where *i* = 1, …, *n*.

Another parameter used is the Akaike information criterion (AIC), which is an extensively used measure for evaluating an ARIMA model. It quantifies the goodness of fit for the model as well as the simplicity of the model. It should be as low as possible [23].

Various measures of goodness of fit, such as RMSE, MAE, MAPE, and AIC, were computed for the ARIMA model. For LSTM, three parameters, RMSE, MAE and MAPE, were calculated.

#### 2.3.1. ARIMA Model

The Autoregressive Integrated Moving Average (ARIMA) is an adaptation of discrete time-filtering methods developed in the 1930–1940s by electrical engineers [15]. Statisticians George Box and Gwilym Jenkins developed systematic methods for applying ARIMA to predict business and economic data in the 1970s. ARIMA is a class of models that capture temporal structures in time series data and employ a linear regression-based forecasting approach. They provide a general framework for the prediction of non-stationary observed time series data [24].

ARIMA combines both auto regression (AR) and moving average (MA) models as well as a differencing pre-processing step of the sequence to make the sequence stationary, which is called integration (I). The notation for the model involves specifying the order of the AR (p means the number of autoregressive terms), I (d means the number of non-seasonal differences), and MA (q means the number of moving-average terms) models as parameters of an ARIMA function. A non-seasonal ARIMA model can be completely summarized by three numbers: p, q and d. *Y* denotes the original series and *y* denotes the differenced series. We can employ the following equations:

$$y_{t}=Y_{t},\;\left(if\;d=0\right);$$

$$y_{t}=Y_{t}-Y_{t-1},\;\left(if\;d=1\right);$$

$$y_{t}=\left(Y_{t}-Y_{t-1}\right)-\left(Y_{t-1}-Y_{t-2}\right)=Y_{t}-2Y_{t-1}+Y_{t-2},\;\left(if\;d=2\right);$$

The forecasting equation for *y* is:

$${\hat{y}}_{t}=\mu +\emptyset_{1}y_{t-1}+\cdots +\emptyset_{p}y_{t-p}-\theta_{1}e_{t-1}-\cdots -\theta_{q}e_{t-q}$$

where μ is a constant, $\emptyset_{1}y_{t-1}\cdots \emptyset_{p}y_{t-p}$ are AR terms (lagged values of *y*), and $\theta_{1}e_{t-1}\cdots \theta_{q}e_{t-q}$ are MA terms (lagged errors).

The seasonal part of an ARIMA model is summarized by three additional numbers: P, D and Q. The complete model is called an “ARIMA (p,d,q)(P,D,Q)” model. p, d and q are orders of auto-regression, the degree of trend difference and the order of moving average, respectively. P, D and Q are seasonal autoregressive terms, seasonal differences and seasonal moving average terms, respectively [9]. For ARIMA (p,d,q)(P,D,Q), we can get:

$$y_{t}=Y_{t}-Y_{t-s},\;\left(if\;d=0,\;D=1\right);$$

$$y_{t}=\left(Y_{t}-Y_{t-1}\right)-\left(Y_{t-s}-Y_{t-s-1}\right)=Y_{t}-Y_{t-1}-Y_{t-s}+Y_{t-s-1},\;\left(if\;d=1,\;D=1\right);$$

where *s* is the seasonal period [25].

ARIMAX is an extension of the ARIMA model, which includes the modeling of exogenous input variables. Exogenous input variables, also known as covariates, can be considered as parallel input sequences, and their observed values have the same time steps as the original series. The original sequence can be used as endogenous data to compare with the exogenous sequences. The observations of exogenous variables are included in the model directly at each time step, and are not modeled in the same way as the primary endogenous sequence (e.g., as an AR, MA, etc. process).

We conducted the ARIMA/ARIMAX analysis in the previous studies [8,19,21,26]. This can be simply described in three stages. These are time series stability, parameter estimation and model evaluation.

The first stage is time series stability. The ARIMA/ARIMAX model requires stationary time series, which means the time series show no fluctuation or periodicity with time. We used the Augmented Dickey–Fuller (ADF) unit-root test to estimate whether the time series is stationary or not. Log transformation and differences are the preferred ways to stabilize the time series. Seasonal differences were adopted to stabilize the term trend and periodicity in this study.

The second stage is parameter estimation. An autocorrelation function (ACF) graph and partial autocorrelation (PACF) graph were used to identify the optimal ARIMA model. Automatic identification and artificial estimation were adopted in this study. The “autoarima ()” command in R software (R Foundation for Statistical Computing, Vienna, Austria) was first adopted to automatically identify the model parameters. Then, ACF, PACF and differences were employed to identify p, d and q and P, D and Q of a seasonal ARIMA model.

The last stage is model evaluation. All the models that passed the Box–Ljung test were compared using AIC and the RMSE of the test set, so that the best model, which usually has the lowest values of AIC and RMSE, can be found.

#### 2.3.2. LSTM Model

**Figure 2:**
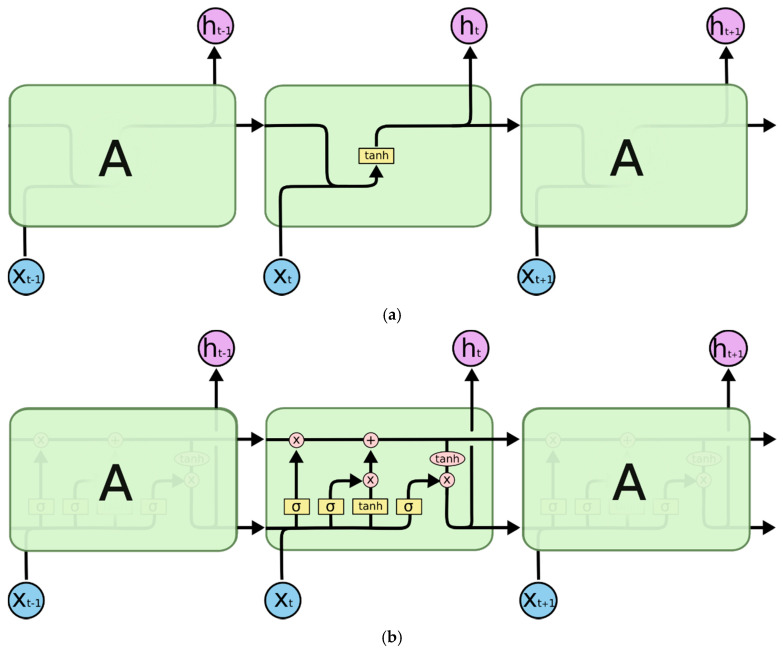
The repeating modules of RNN and LSTM. (**a**). The repeating module in a standard RNN contains a single layer; (**b**). The repeating module in an LSTM contains four interacting layers (A is a chunk of neural network).

Recurrent neural networks (RNNs) are a kind of artificial neural network. They add additional weight to the network and create cycles in the network graph to maintain an internal state. The LSTM Deep Learning algorithm, developed by Hochreiter and Schmidhuber (1997) [26], allows the preservation of weights that are forward- and backpropagated through layers. All recurrent neural networks take the form of a chain of repeating modules of a neural network. In standard RNNs, this repeating module will have a very simple structure, such as a single tanh layer (See Figure 2a) [27]. LSTMs also have this kind of chain-like structure, but the repeating module has a different structure. There is no single neural network layer, but there are four layers that interact in a very special way. In Figure 2b, each line carries an entire vector, from the output of one node to the inputs of others. The pink circles represent pointwise operations, such as vector addition, while the yellow boxes are learned neural network layers. Lines merging denote concatenation, while a line forking denote its content being copied and the copies going to different locations [27]. 

The network can continue to learn over many time steps by maintaining a more constant error. Thus, the network can be used to learn long-term dependencies [26]. LSTM networks try to combat the vanishing/exploding gradient problems by introducing gates and an explicitly defined memory cell. These are inspired mostly by circuitry, not so much by biology. Each neuron contains one memory cell and three gates: input, output and forget. The function of these gates is to safeguard the information by stopping or allowing the flow of it. The input gate determines how much of the information from the previous layer gets stored in the cell. The output layer takes up the job on the other end and determines how much of the next layer gets to know about the state of this cell. The forget gate is useful to forget some prior values, i.e., it controls the extent to which a value remains in the cells due to some future works [26].

LSTM is able to almost seamlessly model problems with multiple input variables. This is a great benefit in time series forecasting, where classical linear methods can be difficult to adapt to multivariate or multiple input forecasting problems.

The original LSTM model consists of a single hidden LSTM layer and a standard feedforward output layer. The Stacked LSTM is an extension to this model. It has multiple hidden LSTM layers, each of which contains multiple memory cells. A Stacked LSTM architecture can be defined as an LSTM model comprised of multiple LSTM layers. The upper LSTM layer provides a sequence output to the lower LSTM layer instead of a single-value output. Stacked LSTM is a stable technique for challenging sequence prediction problems, which makes the model more accurate. In our study, we used a three-layer stacked LSTM to fit the data.

**Figure 3:**
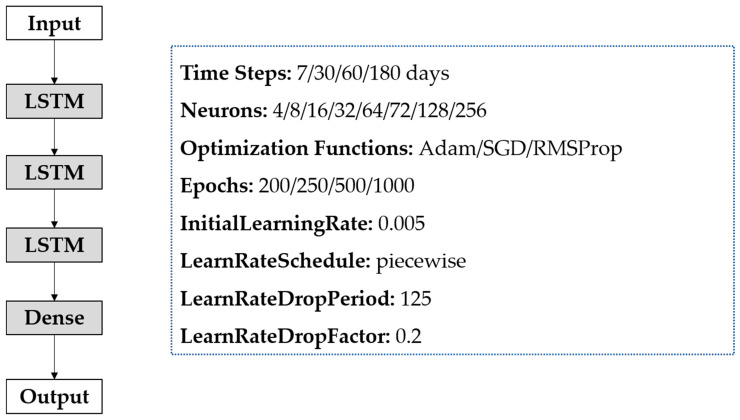
A three-layer stacked Long Short-Term Memory Neural Network (LSTM) architecture.

The training and prediction of the LSTM model can be divided into the following three steps. Firstly, the data were rescaled and normalized to the range of 0 to 1 as the LSTM models were sensitive to the scale of the input data. Secondly, the time steps of univariate and multivariate LSTM were set to 7/30/60/180, which means that we used the data of the previous 7/30/60/180 days to predict the incidence of the next day. Finally, a three-layer stacked LSTM structure was established. Every LSTM layer has one hidden layer that was set for the LSTM model, with neurons options of 4/8/16/32/64/72/128/256. The alternative optimization functions are Adaptive Moment Estimation (Adam), Stochastic Gradient Descent (SGD) and Root Mean Square Prop (RMSProp). All these learning processes were run in 200/250/500/1000 epochs. We specified the initial learning rate as 0.005 and instructed the model to drop the learning rate every 125 epochs by multiplying by 0.2 (See Figure 3). Based on the above results, we chose the optimal model according to the minimum RMSE of the test set.

#### 2.3.3. One Step Ahead Rolling Forecast

In the real world, the environment is unstable and changes quickly. If a disease prediction model cannot respond quickly to changes, then the model is unqualified. Therefore, a flexible prediction model is very important to meeting the challenge. In this study, a rolling forecast scenario, also known as walk-forward model validation, was used.

Each time step of the test dataset is walked one step at a time. The model is used to make a forecast for the time step, and then the actual observed value is obtained from the test set and provided to the model for prediction in the next time step [9]. This mimics a real-world scenario where new daily incidence observations can be obtained every day and used for the prediction of the next day. Through rolling forecast, we can keep abreast of the pulse of the changing situation at any time, and quickly adjust the disease prevention and control points. Therefore, the one step ahead rolling forward forecast is more precise.

The geographical location of Ningbo and Yinzhou station was determined using ArcGIS (version 10.6, ESRI, Redlands, CA, USA). Excel(version 2016, Microsoft, Redmond, WA, USA) was used to build the database of the daily incidence of HFMD in Ningbo, and the ARIMA model and LSTM model were developed by the R software (version 3.6.2, R Foundation for Statistical Computing, Vienna, Austria) with packages “forecast” and “tensorflow”. The significance level is 0.05.

## 3. Results

### 3.1. Descriptive Analysis

Descriptive statistics for the daily incidence of HFMD (incidence) and meteorological variables including Tmean, Pmean, RHmean, WSmean, PPTN and Sunshine are summarized in Table 1. A total of 129,897 HFMD cases from January 2014 to December 2017 were included in our analyses. The daily mean incidence was 88.9 cases. The mean value of Tmean was 17.5 ℃ and its range was from −4.5 ℃ to 32.9 ℃. The mean value of Pmean was 1016.0 hPa and its range was from 985.7 hPa to 1039.7 hPa. The mean value of RHmean was 79.8% and its range was from 34.0% to 100.0%. The mean value of WSmean was 2.0 m/s and its range was from 0.1 m/s to 8.3 m/s. The mean value of PPTN was 5.0 mm and its range was from 0.0 mm to 276.2 mm. The mean value of Sunshine was 4.4 h and its range was from 0.0 h to 12.7 h.

**Figure 4:**
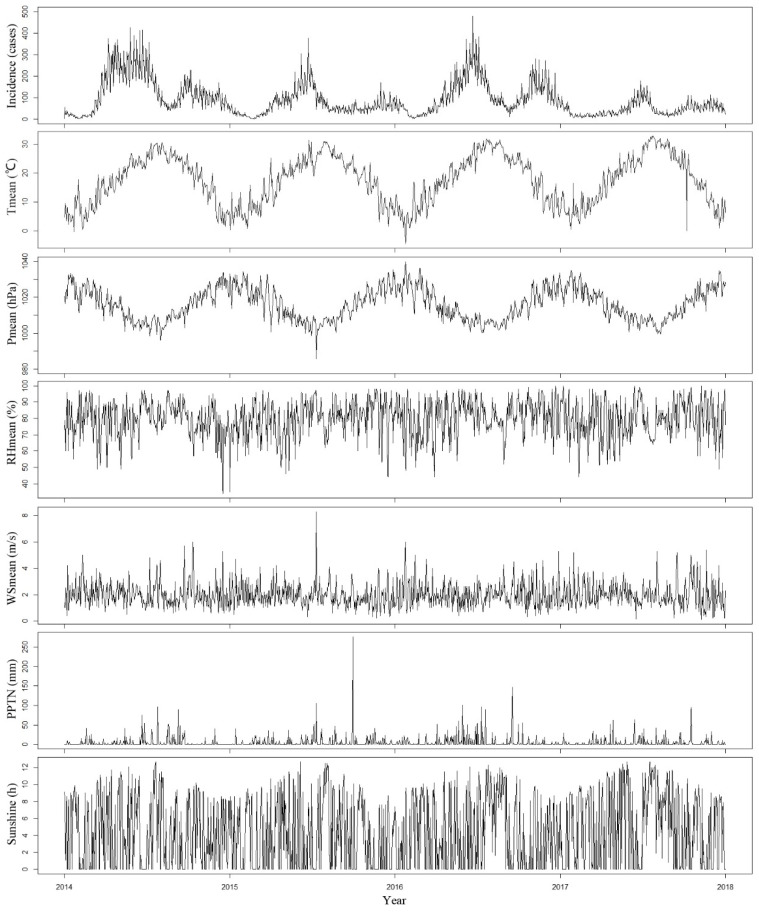
The time series distribution of the daily incidence of HFMD and meteorological variables in Ningbo, 2014–2017.

Figure 4 shows the time series of the daily incidence of HFMD and all of the meteorological variables during the study period. The daily incidence of HFMD in Ningbo exhibits strong seasonality. A bimodal seasonal pattern was observed, which was characterized by peaks in HFMD incidence in the summer (June) and early winter (November).

The univariate Spearman correlation analysis indicated that Tmean, Pmean and RHmean were significantly associated with the incidence of HFMD. Notably, strong correlations were detected between Tmean and Pmean, with correlation coefficients of −0.89 (Table 2). To avoid multicollinearity, only Tmean and RHmean were considered as meteorological variables in the models.

### 3.2. ARIMA and ARIMAX Model

The incidence data of HFMD in Ningbo from January 2014 to November 2017 were used as a training dataset to build prediction models. The time series of the training dataset was not stationary according the result of the ADF test (*p* = 0.10). The first trend difference (d = 1) was assessed to eliminate numerical instabilities in the time series. After the one order differencing, the time series passed the ADF test (*p* = 0.01). Eight alternative ARIMA models (Table 3) with and without external variables (daily mean temperature and daily mean pressure) were primarily selected for further model selection by observing ACF and PACF graphs (Figure S1) and running autoarima () in the R 3.6.2 software. The results of the Box–Ljung test and AIC values of different models are shown in Table 3. 

According to Table 3, all models meet the requirement of white noise for the residual time series (*p* > 0.05), so the AIC values were compared. ARIMA (2,1,1)(0,1,0)_365_ was selected as the best model by autoarima (), as it had the lowest AIC (AIC = 11,439.60). However, it did not perform well in the fitting process of testing (RMSE = 43.27). Therefore, we chose ARIMA (5,1,4) as the best model, because it had the lowest RMSE in the process of testing (RMSE = 12.43). ARIMAX (5,1,4) was selected as best model by autoarima (), which had the lowest AIC (AIC = 13,808.40) and performed well in the process of testing (RMSE = 14.73). The results of the Box–Ljung test for both selected fitting models showed that the residuals satisfied an independent normal distribution (Table 3), which indicated that the fitting models were effective.

### 3.3. Univariate LSTM and Multivariable LSTM Model

Ten alternative univariate LSTM models and ten multivariate LSTM models were listed in Table 4. The results show that the model with 64 neurons and the SGD of univariate LSTM had the lowest RMSE for test set (RMSE = 11.20) in comparison with the models using other parameters. The model with 32 neurons and the Adam of multivariate LSTM had the lowest RMSE for the test set (RMSE = 10.78) in comparison with models using other parameters.

### 3.4. Prediction Performance Comparison

**Figure 5:**
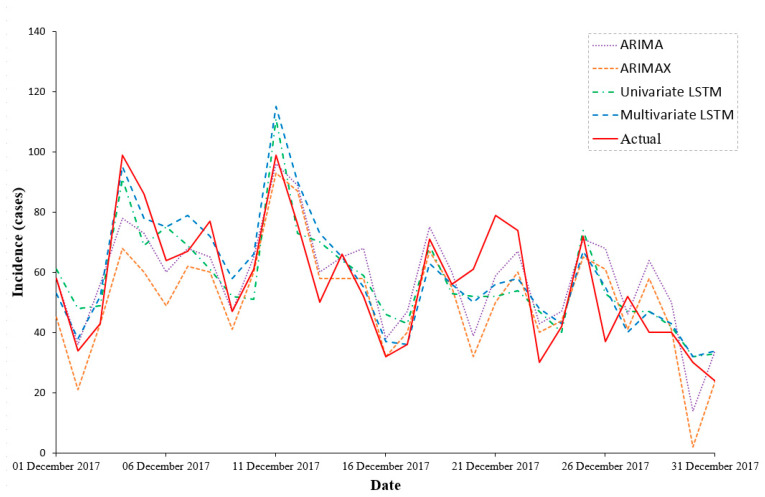
The actual daily incidence of HFMD and values predicted by the four models in December 2017.

The predicting outputs are displayed in Table 5 and Figure 5. Among the four models, the multivariate LSTM model performed best in the prospective forecasting of HFMD incidence over the following 31 days, with the smallest values of RMSE (10.78), MAE (8.71) and MAPE (0.17). The ARIMA showed a better goodness of fit than the ARIMAX model. For the forecast accuracy, the ARIMA model showed a smaller RMSE (12.43) than the ARIMAX model (14.73), as well as a smaller MAE (9.71 vs. 11.26) and MAPE (0.20 vs. 0.21). However, both performed much worse than the two LSTM models.

## 4. Discussion

In this study, the daily mean temperature and daily mean pressure were used as exogenous variables, and four models were used to predict the daily incidence of HFMD. By comparing the results, we found that both of the two LSTM models performed better than the two ARIMA models. This indicates that the LSTM models are more suitable than ARIMA models in predicting the daily incidence of HFMD in Ningbo. At the same time, we found that the prediction performance of the multivariate LSTM model was better than that of the LSTM model without exogenous variables, while the prediction performance of the ARIMA model was better than that of the ARIMAX model. Our findings have profound implications for the local public health departments in terms of establishing precision measures to prevent and control the prevalence of HFMD. 

The two ARIMA models transformed the influence factors of HFMD into some special time variables and then matched them. The limitation of these two ARIMA models is that they can only analyze the linear part of the infectious disease series. However, the non-linear part of the infectious disease data may not be white noise, which means some information may not be captured by the two ARIMA models. LSTM is an advanced kind of Recurrent Neural Network (RNN) and a deep learning application that is designed to learn temporal patterns, capture non-linear dependences, and store useful memory for a longer time, so it produces better results in situations where the number of datasets is large [26]. This may explain why the performances of the two ARIMA models were not as accurate as those of the two LSTM models. The finding that LSTM achieved higher accuracy than ARIMA is also consistent with previous studies [19,28].

Our results indicate that meteorological factors could improve the prediction accuracy of LSTM. Meteorological factors could affect the incidence of HFMD by influencing the breeding, growth, and transmission of pathogens, as well as human behaviors [29,30,31]. Many previous studies have shed light on the non-linear effects of meteorological factors on HFMD [32,33,34,35]. According to the principle of the neural network model, LSTM can effectively fit these non-linear meteorological data. Therefore, the prediction accuracy of multivariate LSTM as regards the influence of meteorological factors was better than that of univariate LSTM without exogenous variables.

As mentioned above, meteorological factors are usually nonlinearly associated with an epidemic of HFMD. Both ARIMA and ARIMAX are essentially linear methods. Using random noise is not very effective in interpreting multiple periodic structures with characteristic fluctuations caused by nonlinear dynamics. This may explain why, compared with the ARIMA model, meteorological factors did not improve the prediction accuracy of ARIMAX, but became interference factors. 

In terms of computational time, the ARIMA models consume more time when using the rolling forecast method, and it is unfeasible to train new models when the orders of p, d and q increase [9]. LTSM models take significantly less time to train, and once trained, constant predictions can be obtained, while ARIMA models need to be retrained. To sum up, LSTM models are more suitable than ARIMA and ARIMAX models for predicting the daily incidence of HFMD in Ningbo.

However, LSTM also has some disadvantages. The optimization of the neural network model is a very complex technical issue. The LSTM model involves the risk of over-fitting or under-fitting, which usually causes poor prediction performance [36]. According to the results, the performance deteriorated when the number of memory cells was less than 32, which suggested that too few memory cells may cause severe under-fit. Moreover, the performance can be improved by increasing the capacity of the model, such as the number of memory cells or number of hidden layers. In addition, when the training epoch is more than 250, the performance of the model will get worse too. Additionally, this phenomenon indicates over-fit. In this study, the selection of the parameter was based on the value of RMSE, and the LSTM model with the minimum RMSE on the test set was selected as the most optimal model.

Admittedly, this study has some limitations. First, the data of the study come from the Data Center of China Public Health Science, which is derived from the hospital reports of HFMD cases. There may be selection or under-reporting bias, which may affect the precision of the predictions. Second, the incidence of HMFD is affected by many natural, social and environmental factors, including the pathogen, environment, host factors, meteorological and air pollution indicators, etc. Given the availability of the data, only meteorological indicators were considered in the model. Third, our study focused on Ningbo, a southern city in China. The epidemiological characteristics of cities in different geographical locations are different, and the associations between the incidence of HFMD and meteorological variables varied with different cities. Therefore, the findings need to be verified when applied to other cities or regions. However, our results warrant further research on the prediction of the daily incidence of HFMD, and provide a scientific reference for the planning of the control and prevention of HFMD in Ningbo.

## 5. Conclusions

In this study, four models were constructed to forecast the daily incidence of HFMD in Ningbo, China. The LSTM model combined with exogenous meteorological variables has the best accuracy among the four models. The results offer a scientific method to build an HFMD early warning system, and help local health departments to make preparations in advance to treat possible outbreaks of HFMD. The methodology could also be applied to other communicable diseases.

## Figures and Tables

**Table 1:** Descriptive statistics of daily incidence of HFMD cases and meteorological factors in Ningbo, 2014–2017.

Indicators	Mean ± SD	Min	P25	P50	P75	Max
Incidence(cases)	88.9 ± 76.8	1	33	64	120	479
Tmean (℃)	17.5 ± 8.4	−4.5	10.1	18.5	24.2	32.9
Pmean (hPa)	1016.0 ± 8.8	985.7	1008.6	1015.7	1023.2	1039.7
RHmean (%)	79.8 ± 11.2	34	73	81	88	100
WSmean (m/s)	2.0 ± 0.9	0.1	1.4	1.8	2.4	8.3
PPTN (mm)	5.0 ± 14.4	0	0	0	3.3	276.2
Sunshine (h)	4.4 ± 4.1	0	0	3.7	8.3	12.7

Note: SD stands for standard deviation, Min stands for minimum value, Max stands for maximum value, P25 stands for 25th percentile, P50 stands for 50th percentile and P75 stands for 75th percentile; Tmean stands for daily mean temperature, Pmean stands for daily mean pressure, RHmean stands for daily mean relative humidity and WSmean stands for daily mean wind speed and PPTN stands for daily precipitation.

**Table 2:** Analysis of correlation between daily incidence of HFMD and meteorological variables.

Indicators	Tmean	Pmean	RHmean	WSmean	PPTN	Sunshine
HFMD	0.34 *	−0.36 *	0.09 *	−0.05	0.04	−0.02
Tmean		−0.89 *	0.15 *	−0.02	0.11 *	0.17 *
Pmean			−0.26 *	0.03	−0.17 *	−0.06 *
RHmean				−0.32 *	0.35 *	−0.58 *
WSmean					0.08 *	0.07 *
PPTN						−0.29 *

Note: *: *p* < 0.05; Tmean stands for daily mean temperature, Pmean stands for daily mean pressure, RHmean stands for daily mean relative humidity and WSmean stands for daily mean wind speed.

**Table 3:** Comparison of the ARIMA and ARIMAX models.

Models	Ljung–Box Test		AIC	RMSE	MAE	MAPE
	X-Squared	*p*-Value				
ARIMA (5,1,4)	2.73	0.10	13,825.48	12.43	9.71	0.21
ARIMA (5,1,2)	0.11	0.74	13,988.18	14.23	11.59	0.24
ARIMA (2,1,1)(0,1,0)_365_	0.02	0.88	11,439.60	43.27	32.59	0.57
ARIMA (3,1,1)(0,1,0)_365_	0.00	0.99	11,440.77	43.2	32.61	0.58
ARIMAX (5,1,3)	0.04	0.84	13,973.31	15.98	12.70	0.22
ARIMAX (4,1,3)	0.97	0.32	14,049.60	17.23	13.49	0.23
ARIMAX (5,1,2)	0.33	0.57	13,973.21	15.92	12.71	0.22
ARIMAX (5,1,4)	3.00	0.08	13,808.40	14.73	11.26	0.21

Note: AIC stands for Akaike information criterion, RMSE stands for root mean square error, MAE stands for mean absolute error and MAPE stands for mean absolute percentage error.

**Table 4:** Comparison of the univariate LSTM and multivariate LSTM models.

Models		Time Steps	Neurons	Optimizer	Epochs	Batch Size	RMSE
Univariate LSTM	1	60	64	SGD	250	32	11.20
	2	60	72	RMSProp	250	16	11.33
	3	60	72	Adam	250	16	11.33
	4	60	72	RMSProp	200	16	11.99
	5	60	72	RMSProp	250	64	12.43
	6	60	64	RMSProp	250	16	12.52
	7	60	128	SGD	250	32	19.30
	8	30	128	SGD	250	32	19.56
	9	180	64	SGD	250	32	20.59
	10	60	32	SGD	250	32	21.57
Multivariate LSTM	1	60	32	Adam	250	32	10.78
	2	60	64	RMSProp	250	32	11.09
	3	60	64	Adam	250	32	11.17
	4	60	64	RMSProp	250	64	12.07
	5	60	64	RMSProp	200	32	12.99
	6	30	32	Adam	250	32	13.64
	7	7	32	Adam	250	32	15.09
	8	180	32	Adam	250	32	15.48
	9	60	128	Adam	250	32	17.07
	10	60	64	SGD	250	32	19.99

Note: SGD stands for stochastic gradient descent, RMSProp stands for root mean square prop, Adam stands for adaptive moment estimation.

**Table 5:** The forecasting performance of the four models.

Model	RMSE	MAE	MAPE
ARIMA (5,1,4)	12.43	9.71	0.20
ARIMAX (5,1,4)	14.73	11.26	0.21
Univariate LSTM	11.20	9.03	0.18
Multivariable LSTM	10.78	8.71	0.17

## Data Availability

Not applicable.

## Supplementary Materials

The following are available online at https://www.mdpi.com/article/10.3390/ijerph18116174/s1, Figure S1: The ACF graph and PACF graph of daily HFMD incidence series.
