# Supplementary figures and images for: Comparison of ARIMA and LSTM in Forecasting the Incidence of HFMD Combined and Uncombined with Exogenous Meteorological Variables in Ningbo, China
Source: Int J Environ Res Public Health. 2021 Jun 7;18(11):6174. doi: 10.3390/ijerph18116174 (PMC8201362; doi:10.3390/ijerph18116174)

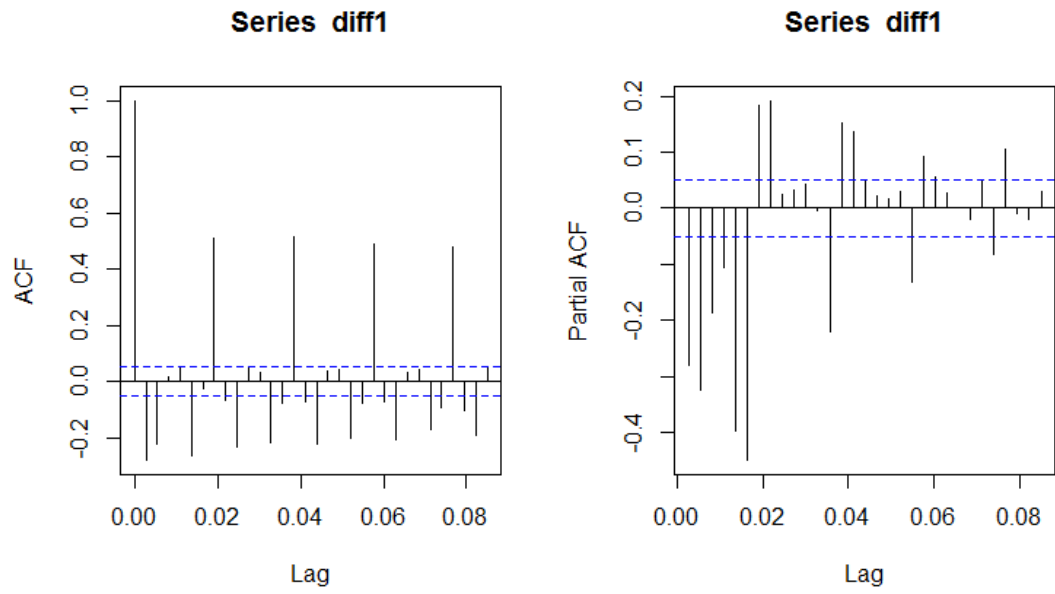

**Figure S1. The ACF graph and PACF graph of daily HFMD incidence series**

Supplement: Supplementary file 1 [file ijerph-18-06174-s001.zip › ijerph-1206138-supplementary.pdf]
